# Polarization-independent regulation of the subcellular localization of Yes-associated protein 1 during preimplantation development

**DOI:** 10.1016/j.jbc.2025.108429

**Published:** 2025-03-19

**Authors:** Shun Saito, Koji Nishiyama, Hanako Bai, Masashi Takahashi, Manabu Kawahara

**Affiliations:** 1 Laboratory of Animal Genetics and Reproduction, Research Faculty of Agriculture, Hokkaido University, Sapporo, Japan; 2 Graduate School of Global Food Resources/Global Center for Food, Land and Water Resources, Hokkaido University, Sapporo, Japan

**Keywords:** cattle, cell differentiation, cell polarity, HIPPO pathway, morula, preimplantation, yes-associated protein 1, zona pellucida

## Abstract

Cell polarization is a crucial developmental process that determines cell differentiation in mouse embryos. During this process, an extensively expressed transcriptional regulator, Yes-associated protein 1 (YAP1), is localized either to the cytoplasm or to the nucleus *via* HIPPO signaling. In mouse premorula embryos, YAP1 is present in the nuclei of all cells. Thereafter, YAP1 is distributed to the nuclei of outer cells or cytoplasm of inner cells, depending on the establishment of cell polarity and morula formation. However, the dynamics of YAP1 localization in other species, including ruminants, remain unclear. To gain an in-depth understanding of cell differentiation in mammalian embryos, we investigated YAP1 localization changes in bovine embryos. Unlike in mouse morulae, YAP1 displayed cytoplasmic localization in most cells, including the outer cells of bovine morulae, after the 32-cell stage. Next, we analyzed the relationship between cell polarity and nuclear localization of YAP1. Polarization of outer cells in the bovine morula began at the late 16-cell stage and was established by the late 32-cell stage, indicating that polarization preceded the nuclear localization of YAP1 in bovine embryos. To explore the regulation of YAP1 localization in bovine morula, we analyzed zona-free embryos and found that the presence of the zona pellucida significantly enhanced YAP1 cytoplasmic localization. Moreover, we observed ectopic expression of SRY-box transcription factor 2 in zona-free blastocysts, which indicated that cytoplasmic localization of YAP1 was associated with the suppression of pluripotency in the trophectoderm. These findings provide valuable insights into the molecular mechanisms underlying the first cell differentiation in mammalian embryos.

The first cell fate decision in mammalian embryos occurs at the morula stage (1, 2). The switching of subcellular localization of Yes-associated protein 1 (YAP1) depending on the cell position constitutes a representative mechanism underlying cell differentiation (3). In outer cells, nuclear localization of YAP1 is promoted by polarization during compaction at the morula stage (4), which contributes to caudal type homeobox 2 (CDX2) expression, and in turn, to trophectoderm (TE) differentiation (3, 5, 6). In inner cells, cytoplasmic localization of YAP1 is induced *via* the activated HIPPO pathway (7, 8), which is important for maintaining the pluripotency of the inner cell mass (ICM). The control mechanisms involved in the nucleocytoplasmic shuttling of YAP1 have been well researched in mouse morulae but not in other species. Especially in bovine, there may be a specific regulatory mechanism underlying YAP1 localization, as suggested in previous reports (9, 10). Both the cell number at the morula stage and the onset time of morula compaction following fertilization constitute species-specific aspects (11, 12). Therefore, further detailed studies on nonrodent species are required to comprehensively understand mammalian embryonic development.

Bovine embryos generally reach the morula stage approximately 5 days after fertilization, or at the 16 to 32 cell stage. Subsequently, blastocoel formation begins approximately 6.5 days after fertilization or at the 80-cell stage (13). The duration of the morula stage in bovine embryos is relatively longer than that in mouse embryos. Thus, the investigation of YAP1 localization at multiple time points during morula formation may be useful for further understanding the mode of differentiation of bovine embryos into the ICM and TE.

Cell polarization is a process that determines the localization of YAP1 in mouse morula (7, 14). In cattle, cell polarity is established during the morula stage (1). However, the precise timing of polarization, including its onset and completion, remains unclear in cattle. Moreover, the role of cell polarity in the subcellular localization of YAP1 in bovine embryos has not yet been elucidated.

The zona pellucida, a crucial structure formed during preimplantation development, provides embryonic cells with appropriate cell contact, correct packing, and intercellular adhesion (15, 16, 17). Abnormal packing of cells after removal of the zona pellucida disrupts morula formation in mouse embryos (15). Disruption of intercellular adhesion by embryo deformation causes abnormal localization of YAP1 in the mouse morula (4). Therefore, removal of the zona pellucida may affect YAP1 localization through abnormal cell contact.

Here, we investigated the relationship between cell polarity and subcellular localization of YAP1 during morula formation in bovine embryos. To account for the variability in developmental progress among bovine embryos, we carefully performed immunostaining by categorizing the examined embryos according to cell number or days postfertilization. Both cell number and culture duration serve as reliable indicators of developmental progress. We found a bovine-specific regulation of YAP1 subcellular localization, demonstrating the significance of the zona pellucida for the subcellular localization of YAP1 during morula formation. Taken together, this study clearly demonstrates that, in bovine embryos, the subcellular localization of YAP1 is independent of cell polarization and the presence of the zona pellucida is required for its precise regulation. This underscores species-specific differences in the regulation of cell fate decisions during preimplantation.

## Results

### Comparison of subcellular localization of YAP1 in bovine and mouse morulae

**Figure 1:**
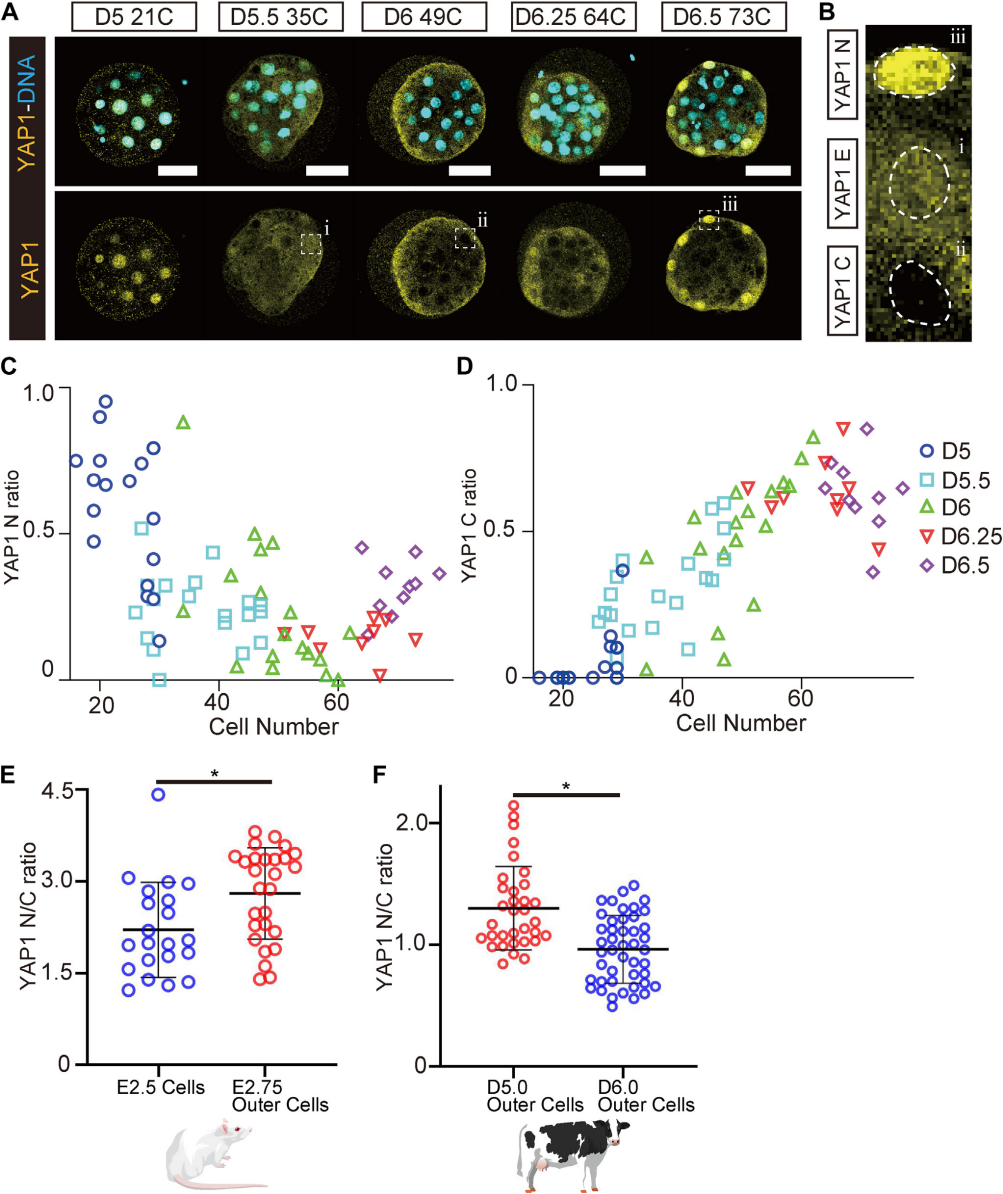
**Localization of YAP1 in bovine morula is relatively different from that in mouse morula.***A*, representative images of YAP1 immunostaining in the bovine morula on D5 (*N* = 18), D5.5 (*N* = 19), D6 (*N* = 18), D6.25 (*N* = 9), and D6.5 (*N* = 10). Capital “C” in the boxes above each image indicates the cell number of the indicated embryo. Scale bars represent 50 μm. (i), (ii), and (iii): *Dashed lines* indicate the cells that are enlarged in *B*. *B*, classification of cells based on the localization of YAP1. *Dashed lines* indicate nuclei. We measured YAP1 N/C ratio in each cell categorized to YAP1 N, E, and C using D5 and D5.5 embryos with relatively large numbers of YAP1 E cells (Fig. S1*B*). Based on this analysis, the thresholds of the N/C ratio were defined as follows (≥1.2: YAP1 N; 0.85–1.2: YAP1 E; and <0.85: YAP1 C). *C*, relationship between the ratio of YAP1 N cells to the total cell number in an embryo (YAP1 N ratio) and the total cell number in the bovine morula. *D*, relationship between the ratio of YAP1 C cells to total cell number in an embryo (YAP1 C ratio) and total cell number in the bovine morula. *E*, comparison of YAP1 N/C ratio between cells in mouse E2.5 embryos (*N* = 3, *n* = 21) and in outer cells in mouse E2,75 embryos (*N* = 3, *n* = 27). *F*, comparison of YAP1 N/C ratio between outer cells in bovine D5.0 embryos (*N* = 3, *n* = 34) and in outer cells in bovine D6.0 embryos (*N* = 3, *n* = 45). Data are represented as the mean ± SD. *Asterisks* indicate significant differences (*p* < 0.05). Statistical differences between samples were analyzed using the Student’s *t* test. Capital “*N*” indicates the number of embryos, and small letter “*n*” indicates the number of cells. N/C, nuclear/cytoplasmic; YAP1, Yes-associated protein 1.

To precisely understand the changes in the subcellular localization of YAP1 during morula formation in bovine embryos, we performed immunostaining for YAP1 using bovine morulae (*n* = 74) with various cell numbers (16–73 cells) and at several time points after fertilization (D5–D6.5) (Figs. 1*A*, S1*A*). The embryonic cells were categorized into three types based on the localization pattern of YAP1: YAP1 N, nuclear localization; YAP1 C, cytoplasmic localization; and YAP1 E, equal localization in both the nucleus and cytoplasm (Fig. 1*B*). To define the categories of YAP1 localization, we measured YAP1 nuclear/cytoplasmic (N/C) ratio in YAP1 N/C/E–classified cells from D5 and D5.5 embryos, which comprise a higher number of YAP1 E cells (Fig. S1*B*). Based on this analysis, the thresholds of the N/C ratio analyzed in the following experiments were defined as follows (≥1.2: YAP1 N; 0.85–1.2: YAP1 E; and <0.85: YAP1 C). In addition, we measured the ratio of each of the three cell types relative to the total number of cells per embryo (Table 1). In D5 embryos, the ratio of YAP1 N cells was 0.597, whereas that of YAP1 C cells was only 0.050 (Table 1). On D5.5, the ratio of YAP1 E cells was 0.451, which was the highest among all the time points (Fig. S1*C*) and the ratio of YAP1 C cells increased when compared with that on D5 (Table 1). On D6 and D6.25, the ratios of YAP1 C cells were 0.476 and 0.633 (Table 1), and the corresponding ratio of YAP1 N cells decreased to 0.233 and 0.143, respectively (Table 1). The outer cells in some embryos showed cytoplasmic localization of YAP1 (Fig. S1*D*), and no YAP1 N cells were observed (Fig. 1*C*). The ratio of YAP1 N cells increased to 0.318 in D6.5 embryos (Table 1), with more intense fluorescence signals in the outer cells than in younger embryos (Fig. 1*A*).

**Table 1:** Ratio of cells classified by YAP1 localization in bovine and mouse morula

Culture duration	YAP1 N ratio (YAP1 nuclear cell/total cell)	YAP1 E ratio (YAP1 equal cell/total cell)	YAP1 C ratio (YAP1 cytoplasmic cell/total cell)
Bovine			
D5	0.597 ± 0.233	0.353 ± 0.169	0.050 ± 0.092
D5.5	0.241 ± 0.122	0.451 ± 0.160	0.308 ± 0.149
D6	0.233 ± 0.228	0.291 ± 0.150	0.476 ± 0.226
D6.25	0.143 ± 0.060	0.224 ± 0.093	0.633 ± 0.113
D6.5	0.318 ± 0.094	0.105 ± 0.102	0.577 ± 0.113
Mouse			
E2.75	0.868 ± 0.104	0.089 ± 0.095	0.048 ± 0.068
E3.25	0.666 ± 0.121	0.131 ± 0.084	0.202 ± 0.099
E3.5	0.674 ± 0.095	0.175 ± 0.155	0.151 ± 0.110

Results are expressed as mean ± SD.

To compare subcellular localization of YAP1 in bovine morulae with that in mouse morulae, we analyzed the subcellular localization in 35 mouse morula embryos (Fig. S1*E*, Table 1). In mouse morulae, the YAP1 N ratio per embryo was not less than 0.473 (Fig. S1*F*), and the YAP1 C ratio was not more than 0.400 (Fig. S1*G*). Compared with mouse morulae, cytoplasmic localization of YAP1 was more pronounced in bovine morulae. Subsequently, we measured the YAP1 N/C ratio in all cells and specifically outer cells in mouse and bovine embryos (Fig. 1, *E* and *F*). YAP1 predominantly localized to the nucleus of most cells in E2.5 mouse morulae and D5 bovine morulae. Therefore, we considered that the E2.5 mouse morulae and D5 bovine morulae were at same developmental stage and compared their YAP1 N/C ratio to embryos in subsequent developmental stages such as that of E2.75 mouse morulae and D6 bovine morulae. In the mouse E2.5 morula, the cell number ranges between six and eight cells with no fully internalized cell; therefore, we measured the ratio across all cells. In the E2.75 mouse morula, the YAP1 N/C ratio was significantly higher than that in E2.5 (Fig. 1*E*). Conversely, the YAP1 N/C ratio of the D6 bovine morula was significantly lower than that in the D5 morula (Fig. 1*F*). These results indicate that YAP1 in the bovine morula localizes to the cytoplasm rather than to the nucleus, unlike in the mouse morula.

### Nuclear localization of YAP1 during morula formation in bovine embryos

**Figure 2:**
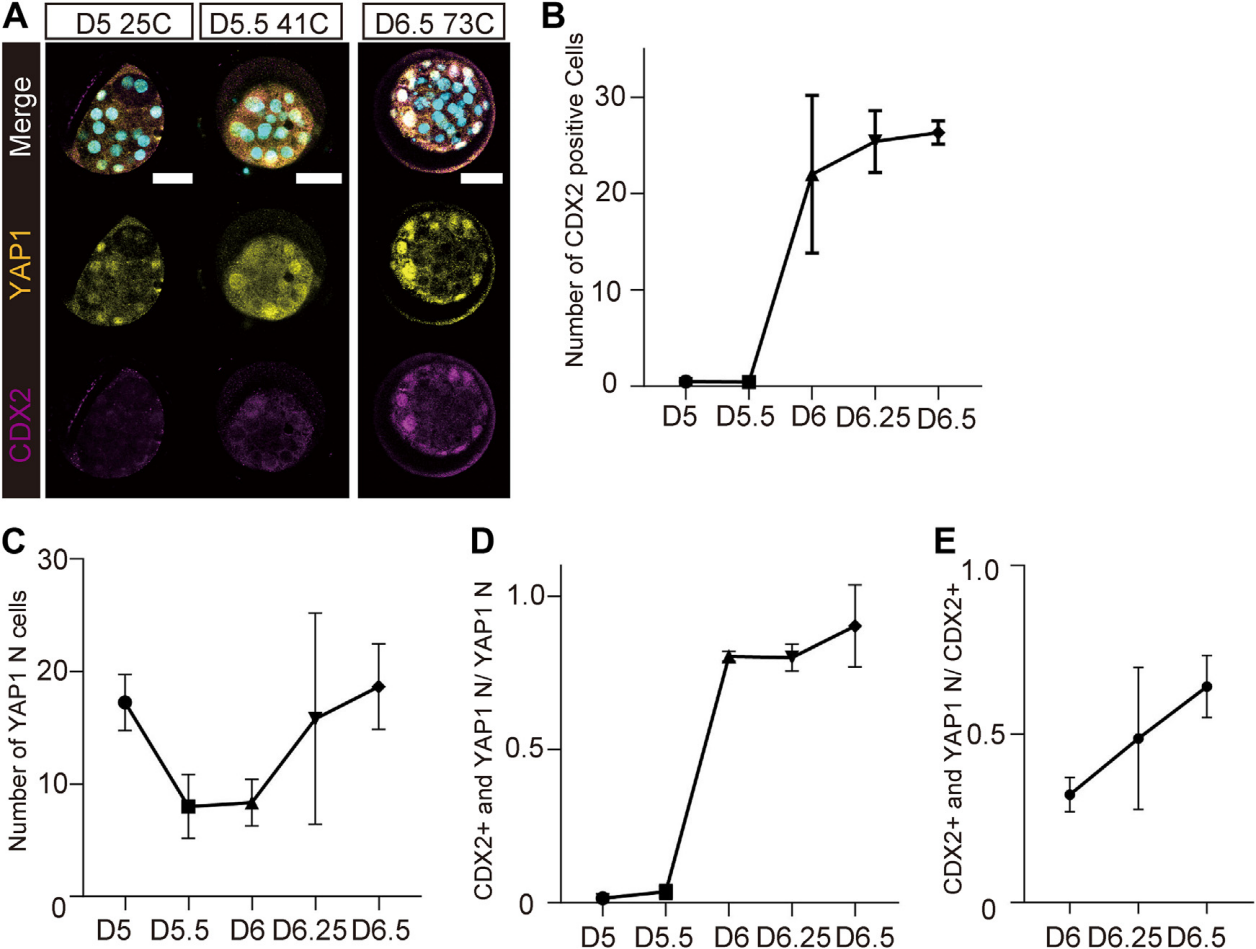
**Relationship between YAP1 localization and CDX2 expression in bovine morula.***A*, immunostaining of YAP1 and CDX2 in the bovine morula on D5, D5.5, and D6.5. Scale bars represent 50 μm. Bovine morulae were used for the following analyses on each sampling day (D5, *N* = 4; D5.5, *N* = 7; D6, *N* = 3; D6.25, *N* = 5; and D6.5, *N* = 3). *B*, number of CDX2-positive cells on D5, D5.5, D6, D6.25, and D6.5. *C*, number of cells in which YAP1 was localized to the nuclei on D5, D5.5, D6, D6.25, and D6.5. *D*, relationship between the ratio of CDX2-positive cells with nuclear localization of YAP1 and all cells with nuclear localization of YAP1 and days after fertilization. *E*, relationship between the ratio of CDX2-positive cells and nuclear localization of YAP1 to all CDX2-positive cells and days after fertilization. Data are represented as the mean ± SD (*B*–*E*). CDX2, caudal type homeobox 2; YAP1, Yes-associated protein 1.

To determine the significance of nuclear localization of YAP1 from the D5 to D6.5 morula stage, we performed immunostaining for both CDX2 and YAP1 (Fig. 2*A*). CDX2 expression was observed on D6 (Fig. 2*B*), whereas YAP1 N cells started increasing on D6.25 (Fig. 2*C*). The nuclear localization of YAP1 in the early morula on D5 or D5.5 was not concomitant with CDX2 expression (Fig. 2*D*). In the late morula stage, the nuclear localization of YAP1 was accompanied by CDX2 expression after D6 (Fig. 2*D*). The ratio of YAP1 N cells expressing CDX2 to CDX2-positive cells increased with developmental progress up to D6.5 (Fig. 2*E*). Unlike in mouse embryos, the expression of CDX2 preceded the nuclear localization of YAP1 in the early morula stage of bovine embryos, although YAP1 nuclear localization synchronized with CDX2 expression in the late morula stage up to D6.5.

We further investigated the ratio of TE cells with CDX2 expression in bovine and mouse blastocysts (Fig. S2, *A* and *B*). The ratio of CDX2-positive cells in bovine early blastocysts was significantly lower than that in bovine middle blastocysts and early mouse blastocysts (*p* < 0.05) (Fig. S2*B*). The YAP1 N ratios in D6.5 bovine and mouse E3.5 morulae were evaluated to investigate the cause for the lower CDX2-positive ratio in early bovine blastocysts. The YAP1 N ratio in D6.5 bovine morulae was significantly lower than that in mouse morulae at E3.5 (*p* < 0.05) (Fig. S2*C*). This lowered YAP1 N ratio in bovine D6.5 morulae might be associated with fewer CDX2-positive cells in bovine early blastocysts at D6.5.

### Polarization preceding nuclear localization of YAP1 in bovine morula

**Figure 3:**
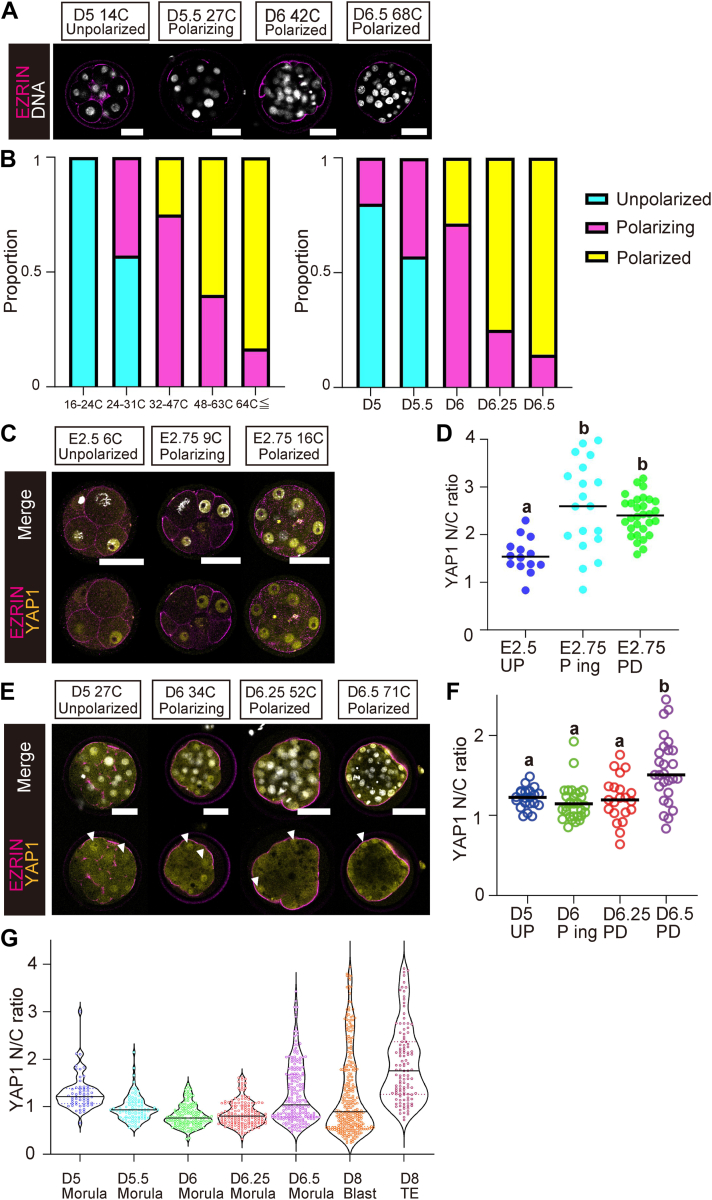
**Effect of cell polarization on nuclear localization of YAP1 in bovine morula.***A*, immunostaining of EZRIN in bovine morula on D5 (*N* = 10), D5.5 (*N* = 7), D6 (*N* = 7), D6.25 (*N* = 8), and D6.5 (*N* = 7). *B*, ratios of unpolarized (UP), polarizing (P ing), and polarized (PD) embryos depend on the number of cells and number of days after fertilization. The number of embryos in each group was <24C embryos (*N* = 4), 24–35C embryos (*N* = 14), 36–47C embryos (*N* = 4), 48–63C embryos (*N* = 5), and ≥64C embryos (*N* = 12). *C*, immunostaining of YAP1 and EZRIN in mouse morula at various cell numbers and days after fertilization. E2.5 UP embryos (*N* = 4), E2.75 P ing embryos (*N* = 4), and E2.75 PD embryos (*N* = 3). *D*, comparison of the YAP1 N/C ratio among UP cells in D2.5 embryos (*N* = 3, *n* = 14), P ing cells in E2.75 embryos (*N* = 3, *n* = 19), and PD cells in E2.75 embryos (*N* = 3, *n* = 30). ^a,b^Different figures indicate statistical significance (*p* < 0.05). *E*, immunostaining of EZRIN and YAP1 in bovine morula at various cell numbers and days after fertilization. *Arrowheads* indicate the cells in which YAP1 was localized to the nucleus. *F*, comparison of the YAP1 N/C ratio among UP cells in D5 embryos (*N* = 3, *n* = 21), P ing cells in D6 embryos (*N* = 3, *n* = 31), PD cells in D6.25 embryos (*N* = 3, *n* = 21), and PD cells in D6.5 embryos (*N* = 3, *n* = 27). Different letters indicate a significant difference (*p* < 0.05). ^a,b^Different figures indicate statistical significance (*p* < 0.05). *G*, comparisons of the YAP1 N/C ratio in each cell among the D5–D6.5 morulae (D5, *n* = 59; D5.5, *n* = 93; D6, *n* = 142; D6.25, *n* = 156; and D6.5, *n* = 204), D8 blastocyst (*n* = 293), and TE (*n* = 115) in D8 blastocyst. Three embryos from each group were used for analysis. Scale bars REPRESENT 50 μm (*A*, *C*, *E*). Capital “*N*” indicates the number of embryos, and small letter “*n*” indicates the number of cells. Statistical significance was analyzed using one-way ANOVA, followed by Dunn’s multiple comparison test (*D*, *F*). N/C, nuclear/cytoplasmic; TE, trophectoderm; YAP1, Yes-associated protein 1.

Cell polarization is a critical process determining the subcellular localization of YAP1 in mouse embryos (14). To determine the timing of polarization in bovine embryos, we first investigated the subcellular localization of the cell polarity–related protein EZRIN using immunostaining. In the D5 morulae, EZRIN was localized around the cell membrane (Fig. 3*A*). On D5.5, EZRIN expression was not observed at the cell adhesion site but was observed at a few non–cell adhesion sites (Fig. 3*A*). These results indicated that the polarity of outer cells began at D5.5 in bovine morulae. On D6, EZRIN expression was observed in a large part of the non–cell adhesion sites in most embryos (Fig. 3*B*), indicating that they underwent polarization and were nearly completely polarized. In most of the D6.25 and D6.5 embryos, EZRIN was expressed throughout the non–cell adhesion site and polarization was established (Fig. 3, *A* and *B*). The apical localization of phospho-EZRIN, RADIXIN, and MOESIN was similar to that of EZRIN in the D6 and D6.5 morulae (Fig. S3); however, in the D5 morulae, the phospho-EZRIN, RADIXIN, and MOESIN signal was only observed at the non–cell adhesion site (Fig. S3). Overall, polarization in bovine morulae starts from D5.5, at the 16-cell stage, and is completed on D6.25, at the 32-cell stage (Fig. 3*B*).

Next, to explore the relationship between the nuclear localization of YAP1 and polarization, we performed immunostaining for YAP1 and EZRIN. The progress of both polarization and YAP1 nuclear localization occurred synchronously in mouse embryos (Fig. 3, *C* and *D*). In contrast, despite the establishment of polarization in the outer cells of D6 and D6.25 bovine morulae, the nuclear localization of YAP1 was not enhanced when compared with that in the D5 morulae (Fig. 3, *E* and *F*). In the D6.5 morulae, the nuclear localization of YAP1 increased in polarized outer cells (Fig. 3, *E* and *F*). In addition, mouse neurofibromin 2-GFP (mNF2-GFP) was localized throughout the cell membrane, and the disappearance of NF2 from the apical domain was not observed in bovine morula (Fig. S3*B*). Cells with a YAP1 N/C ratio equivalent to that of the D8 TE were observed from D6.5 onward (Fig. 3*G*). These results demonstrated that polarization occurred prior to the nuclear localization of YAP1 in the bovine morulae, unlike in the mouse morulae.

### Factors affecting cytoplasmic localization of YAP1 in bovine morula

**Figure 4:**
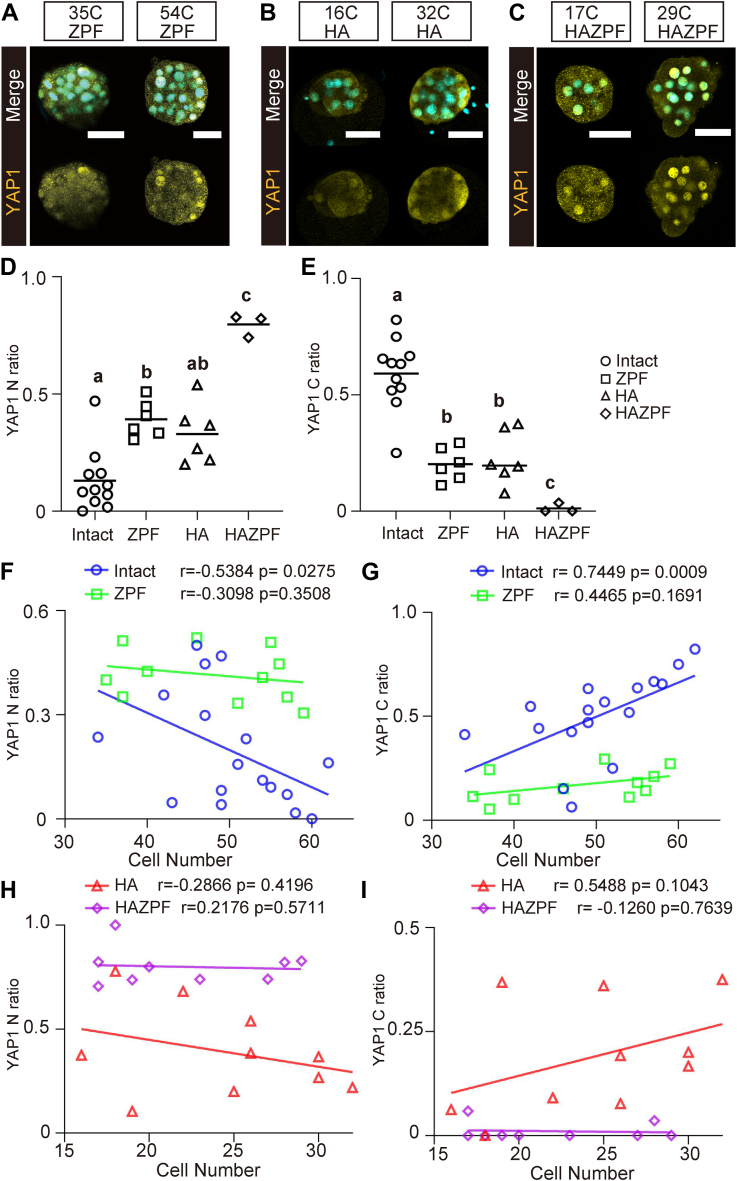
**Effects of the presence of zona pellucida and/or cell number on subcellular localization of YAP1 in bovine embryos.***A*, immunostaining for YAP1 in zona pellucida-free (ZPF) D6 embryos (*N* = 11). *B*, immunostaining of YAP1 in embryos with half the number of cells prepared by destroying either one of the two blastomeres in the 2-cell stage (HA) D6 embryos (*N* = 10). *C*, immunostaining of YAP1 in embryos prepared by blastomere separation after removal of the zona pellucida (HAZPF) D6 embryos (*N* = 19). *D*, comparison of the YAP1 N ratio among intact (*N* = 17), ZPF (*N* = 6), HA (*N* = 6), and HAZPF (*N* = 3) embryos at the late morula stage (intact and ZPF, 48–64 cells; HA and HAZPF, 24–32 cells). ^a–c^Different figures indicate statistical significance (*p* < 0.05). *E*, vomparison of the YAP1 C ratio among intact, ZPF, HA, and HAZPF embryos at the late morula stage. ^a–c^Different figures indicate statistical significance (*p* < 0.05). *F*, relationship between YAP1 N ratio and total cell number in intact and ZPF embryos. *G*, relationships between the YAP1 C ratio and total cell number in intact and ZPF embryos. *H*, relationship between YAP1 N ratio and total cell number in HA and HAZPF embryos. *I*, relationship between the YAP1 C ratio and total cell number in HA and HAZPF embryos. Statistical differences were analyzed using one-way ANOVA, followed by Dunn’s multiple comparison test (*D*, *E*) or nonparametric Spearman’s rank correlation (*F*–*I*). Scale bars represent 50 μm (*A*–*C*). YAP1, Yes-associated protein 1.

**Figure 5:**
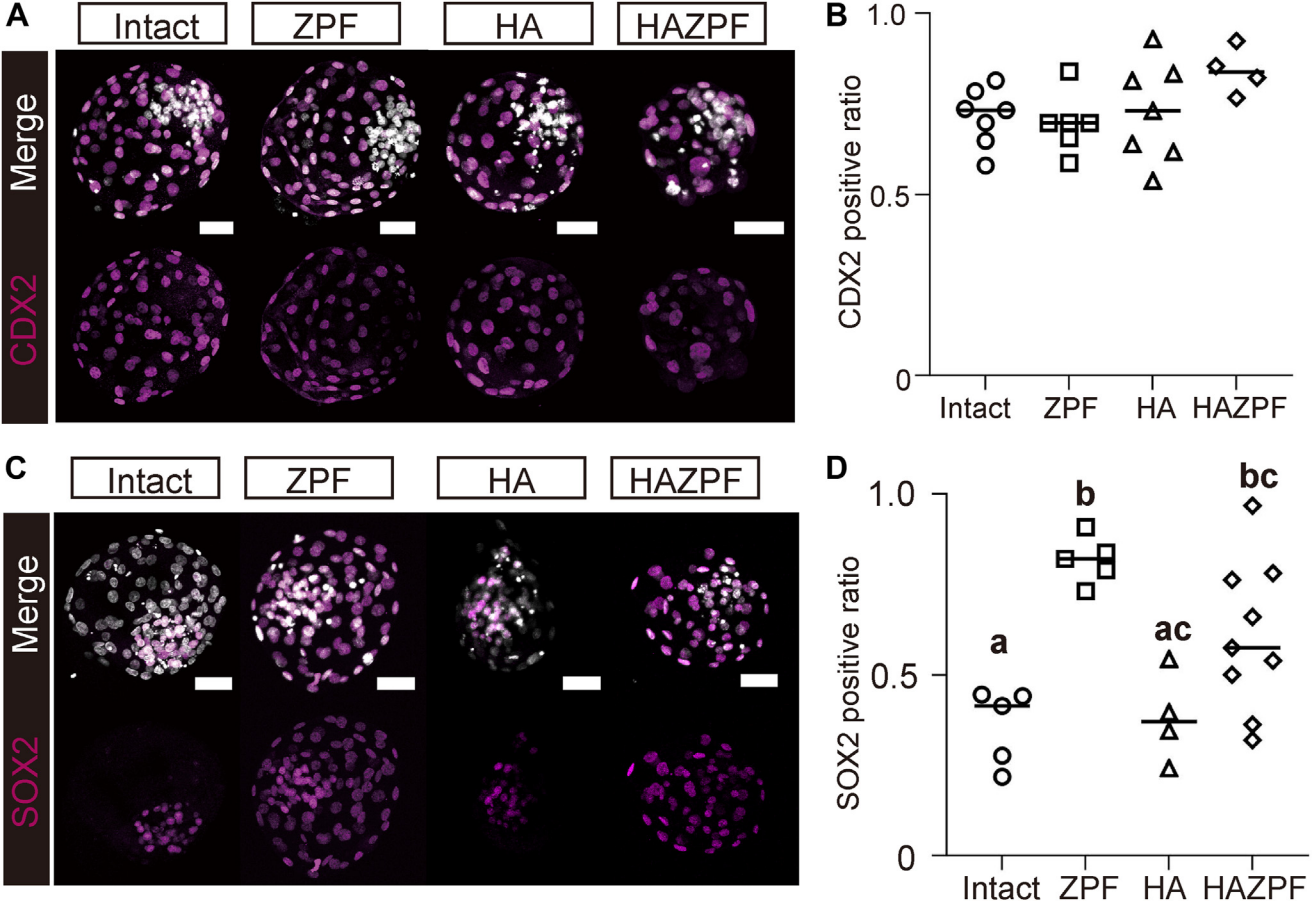
**Effects of the presence of zona pellucida and/or cell number on the expression of CDX2 and SOX2 in bovine embryos.***A*, immunostaining of CDX2 in intact (*N* = 7), zona pellucida-free (ZPF) (*N* = 6), embryos with half the number of cells prepared by destroying either one of the two blastomeres at the 2-cell stage (HA) (*N* = 7), and embryos prepared by blastomere separation after removal of the zona pellucida (HAZPF) (*N* = 4) D8 blastocyst. *B*, comparison of the ratio of CDX2-positive cells to the total cell number per embryo among intact, ZPF, HA, and HAZPF D8 blastocyst. *C*, immunostaining for SOX2 in intact (*N* = 5), ZPF (*N* = 5), HA (*N* = 4), and HAZPF (*N* = 9) D8 blastocysts. *D*, comparison of the ratio of SOX2-positive cells to the total cell number per embryo among intact, ZPF, HA, and HAZPF D8 blastocyst. ^a,b^Different figures indicate statistical significance (*p* < 0.05). Statistical differences were analyzed using one-way ANOVA followed by Dunn’s multiple comparison test (*B*, *D*). Scale bars represent 50 μm (*A*, *C*). CDX2, caudal type homeobox 2; SOX2, SRY-box transcription factor 2.

To investigate how the subcellular localization of YAP1 is regulated in bovine morulae, we prepared three types of embryos: zona pellucida–free embryos (ZPF), embryos with half the number of cells by destroying either one of the two blastomeres at the 2-cell stage (HA), and embryos prepared by separation of blastomeres after removal of the zona pellucida (HAZPF) (Fig. S4*A*). These embryos underwent immunostaining for YAP1 on D6 (Fig. 4, *A*–*C*). The YAP1 N ratio significantly increased in ZPF and HAZPF embryos (*p* < 0.05) (Fig. 4*D*), whereas the YAP1 C ratio significantly decreased in the late morula stage (*p* < 0.05) (Fig. 4*E*) when compared with that in the intact controls. Apical localization of EZRIN was not inhibited by zona pellucida removal (Fig. S4*B*). This finding indicated the lack of association between localization of YAP1 and establishment of polarity in bovine morulae. The YAP1 C ratio significantly decreased in the 24- to 32-cell HA embryos (Fig. 4*E*) but was comparable to the control in ≥32-cell embryos (Fig. S4, *C*–*E*). Furthermore, no significant changes in either the YAP1 N or YAP1 C ratios were observed with an increase in the cell number in ZPF and HAZPF embryos (Fig. 4, *F*–*I*). At the blastocyst stage, the proportion of CDX2-positive cells did not change in any of the embryo types (Fig. 5, *A* and *B*). However, ectopic expression of the pluripotency-related transcription factor, SRY-box transcription factor 2 (SOX2), was observed in the TE (Fig. 5*C*), and the ratio of SOX2-positive cells significantly increased in the ZPF and HAZPF embryos (*p* < 0.05) (Fig. 5*D*). To address the association between nuclear localization of YAP1 and SOX2 expression, we performed immunostaining of SOX2 and YAP1 in both intact and ZPF bovine morulae. SOX2 expression was detected in YAP1 N cells in intact D5, D6, and ZPF D6 morulae (Fig. S5, *A*–*C*). In addition, SOX2 expression in YAP1 N cells of intact D5 and D6 morulae (Fig. S5, *A* and *B*) was weaker when compared with that in YAP1 N cells of D6 ZPF morulae (Fig. S5*C*).

## Discussion

The localization of YAP1, which regulates a broad range of transcripts, contributes to cell fate determination in mouse embryos (3, 18). However, when and how cell-to-cell differences in YAP1 localization determine the cell fate in bovine embryos remain unclear. In this study, we found that the dynamics of YAP1 subcellular localization in bovine morulae was completely different from that in mouse morulae. Furthermore, this unique YAP1 localization in bovine morulae required the presence of the zona pellucida, unlike the strict control through cell polarization in mouse embryos. These findings may reflect the characteristic roles of bovine YAP1 and the regulation of its subcellular localization during preimplantation development.

In bovine morulae, a higher number of cells exhibited cytoplasmic localization of YAP1 when compared with mouse morulae (Table 1). In addition, there was a specific stage during which YAP1 localization was predominantly cytoplasmic across all cells in the embryo (Fig. 1, *E* and *F*). These findings suggest that the enhanced cytoplasmic localization of YAP1 in bovine morula is a distinctive characteristic.

The cytoplasmic localization of YAP1 may be required for morula-specific transcriptome in bovine embryos; however, there are no reports describing its significance in bovine embryos. Notably, the cytoplasmic localization of YAP1 was inhibited, and nuclear localization of YAP1 was maintained in ZPF and HAZPF embryos at the morula stage after removal of the zona pellucida (Fig. 4, *D* and *E*). At the blastocyst stage, ZPF and HAZPF embryos did not express CDX2 in ICM, and instead, expressed a representative pluripotency-related transcription factor, SOX2, in TE (19, 20, 21) (Fig. 5, *C* and *D*). These results indicated that the increasing presence of YAP1 in the cytoplasm in outer cells was crucial for suppressing pluripotency and appropriate transcriptomic profiles of TE cells during preimplantation development in cattle.

In D5 and D6 morulae, SOX2 expression was observed in cells with nuclear localization of YAP1 (Fig. S5, *A* and *B*). In mouse morulae, nuclear YAP1 is essential for repressing SOX2 expression in outer cells (3), whereas in other cell lineages, such as the mouse epiblast, nuclear localization of YAP1 promotes SOX2 expression (22, 23). These findings suggest that regulation of SOX2 expression by YAP1 localization varies spatiotemporally within the embryo. In early bovine morulae, nuclear localization of YAP1 did not appear to inhibit expression of SOX2 (Fig. S5, *A* and *B*). In zona pellucida–free embryos, the relationship, where nuclear localization of YAP1 does not inhibit SOX2 expression, may persist until the blastocyst stage (Figs. 5*C*, S5*C*).

We focused on cell polarization to understand the molecular mechanisms controlling YAP1 localization in bovine embryos, which operates differently from that in mouse embryos. According to microvilli analyses in bovine embryos, the developmental stage for the initiation of cell polarization is around the 16-cell stage (24). However, the timing of cell polarization has not been precisely determined. EZRIN, a representative polarity-related protein, localizes to the apical domains of polarized cells during the morula stage in mice (25). Cell polarity analysis according to EZRIN localization revealed that polarization in bovine embryos began around the 24–31-cell stage on D5.5 and was completed around the 48–63-cell stage on D6.25 in our culture system (Fig. 3, *A* and *B*). These results showed that the establishment of polarity occurred later than estimated based on microvilli formation in the bovine morula. Enhancement of the nuclear localization of YAP1 and establishment of cell polarity were simultaneously initiated in the mouse morula, as described in a previous study (Fig. 4, *C* and *D*) (4). Hence, these results indicate that cell polarization precedes nuclear localization of YAP1 in bovine embryos, unlike in mouse embryos (Fig. 4, *E* and *F*). NF2 is a component of the HIPPO signaling pathway that links its activity to cell polarity (26). In mouse morulae, the loss of NF2 from the apical membrane is crucial for HIPPO signal inactivation (8, 14). In bovine morulae, the loss of NF2 from the apical membrane was not observed until attenuation of mNF2-GFP signal on D5.5 (Fig. S3*B*). This finding suggests that HIPPO signal inactivation in bovine morulae was not triggered by the establishment of cell polarity. In summary, unlike in mouse morulae, the progression of cell polarity formation and nuclear localization of YAP1 were not linked in bovine morulae.

Next, we examined the effects of the zona pellucida and cell number on YAP1 localization in the bovine morula. We prepared three types of embryos, namely the ZPF, HA, and HAZPF embryos. Among these, removal of the zona pellucida in ZPF and HAZPF embryos drastically influenced the subcellular localization of YAP1 in the morula (Fig. 4, *A* and *C*), which inhibited the cytoplasmic localization depending on the increase in cell number (Fig. 4, *D*–*I*). The loss of the zona pellucida in mouse embryos disrupts cell–cell adhesions (15). In mouse morulae, non–cell-adhesive surfaces promoted the nuclear localization of YAP1 (4). Therefore, the maintenance of nuclear localization of YAP1 in bovine ZPF and HAZPF embryos may be due to an increase in non–cell-adhesive surfaces in the absence of zona pellucida.

The YAP1 N ratio in HA embryos remained unaffected, but the YAP1 C ratio decreased in ≤32-cell embryos (Fig. 4, *B* and *E*). The cytoplasmic localization of YAP1 is promoted by decreased cell density in stem and somatic cells (27, 28). The decreased YAP1 C ratio in ≤32-cell HA embryos might be due to decreased cell density. These results suggest that cell density could also impact YAP1 localization, although it was not as critical as the removal of zona pellucida.

Inhibition of the cytoplasmic localization of YAP1 during morula formation leads to ectopic expression of SOX2, which is essential for pluripotency in bovine embryos (29). However, we could not elucidate the detailed molecular mechanism driving the cytoplasmic localization of YAP1 in the bovine morula. In mouse embryos, the subcellular localization of YAP1 regulators, such as ACTIN, AMOT, and LATS2, is also altered at the onset of cell polarity, and HIPPO signaling is turned off to promote the nuclear localization of YAP1 (7, 14, 30, 31). ACTIN localization is critical for controlling YAP1 localization in bovine embryos, as shown in our previous study (32). However, the HIPPO signaling components other than those described previously have not yet been identified in bovine embryos. Therefore, future analysis of the localization of these HIPPO signaling components in bovine embryos will be crucial for elucidating the reason why YAP1 is not localized to the nucleus in bovine embryos despite the initiation of cell polarity formation.

**Figure 6:**
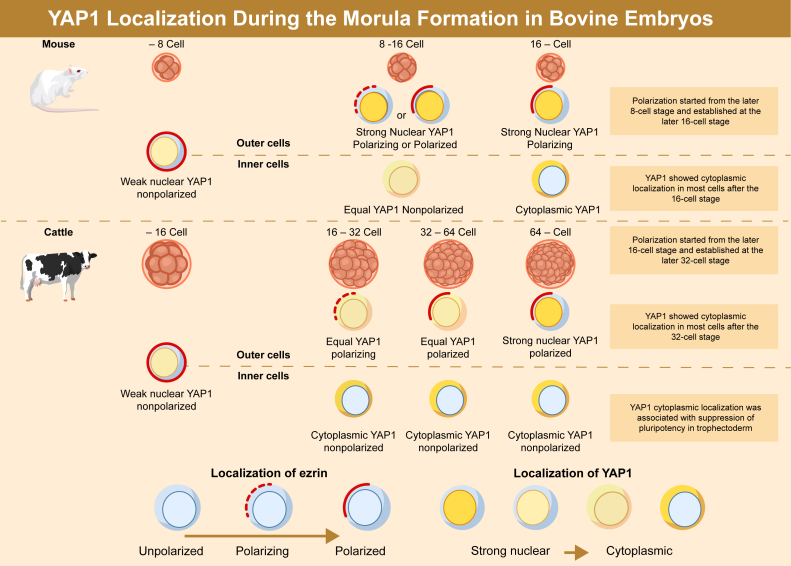
**Difference in the localization of YAP1 and EZRIN between mouse and bovine morulae.** Summary of the results of this study. *Red and dashed lines* along the cell membrane indicate the localization of EZRIN. *Dashed lines* indicate incomplete localization of EZRIN in non–cell adhesion membranes. *Yellow* indicates the localization of YAP1. The *upper panel* indicates the mouse morula model, and the *lower panel* indicates the bovine morula model. YAP1, Yes-associated protein 1.

Overall, our results revealed that the subcellular localization and regulation of YAP1 in the bovine morula were distinct from those in the mouse morula (Fig. 6). In addition, we found that the zona pellucida is important not only for the subcellular localization of YAP1 but also for the first cell differentiation with appropriate SOX2 expression. As the period required for morula formation and cell number differs between cattle and mice, the molecular mechanisms regulating cell differentiation need to be investigated for each species. Our findings provide new insights for a comprehensive understanding of first cell differentiation in mammalian embryos.

## Experimental procedures

### Ethical approval

All animal experiments were approved by the Regulatory Committee for the Care and Use of Animals of Hokkaido University (Approval No.: 24-0103) and were performed in accordance with the National University Corporation Hokkaido University Regulations on Animal Experimentation.

### Preparation of *in vitro* fertilized embryos and sampling

Bovine embryos were prepared *via in vitro* fertilization, as described previously (33, 34). Briefly, cumulus–oocyte complexes (COCs) were aspirated from 3 to 8 mm follicles in ovaries retrieved from a slaughterhouse. COCs, including intact cumulus cells, were cultured at 38.5 °C in a humidified atmosphere with 5% CO_2_ and air for 20 to 22 h in 100 ml droplets of TCM199 (Gibco) containing 10 mM cysteamine (Sigma–Aldrich), 10% (v/v) fetal bovine serum (PAA Laboratories), 0.5 mg/ml follicle-stimulating hormone (Kyoritsu Seiyaku Corp), 100 U/ml penicillin (Nacalai Tesque, Inc), and 100 U/ml streptomycin (Nacalai Tesque, Inc) covered with liquid paraffin oil. The oocytes were then transferred to Brackett and Oliphant medium (35) containing 2.5 mM theophylline (Wako Pure Chemical Industries, Ltd). Frozen-thawed semen was centrifuged at 600*g* for 7 min in Brackett and Oliphant medium, and spermatozoa were added to the COCs at a final concentration of 5 × 10^6^ cells/ml. Following 12 h of incubation, presumptive *in vitro*-fertilized zygotes were denuded by pipetting and cultured at 38.5 °C in a humidified atmosphere with 5% CO_2_ using synthetic oviduct fluid (SOF) medium supplemented with 10 mg/ml insulin (Sigma–Aldrich), until further sampling. The capital “D” in figures indicates the days after fertilization and was determined by setting the start of culture in SOF medium as D0.75.

Mouse embryos were prepared *via in vitro* fertilization, as described previously (36). Briefly, female ICR mice were superovulated 48 h apart *via* intraperitoneal injection of 7.5  IU equine chorionic gonadotropin (ASKA Pharmaceutical Co, Ltd) and 7.5  IU human chorionic gonadotropin (ASKA Pharmaceutical Co, Ltd). Sperms were collected from the cauda epididymis of male ICR mice, suspended in a 200 μl drop of human tubal fluid (HTF) medium in paraffin oil, and preincubated for 90 min in a 5% CO_2_ atmosphere at 37 °C. Oocytes at metaphase II were collected from murine oviducts 16 h after human chorionic gonadotropin administration and transferred to a 100 μl drop of HTF containing 0.5 to 1 × 10^6^ sperm/ml. Embryos were washed with M2 medium 4 to 6 h after insemination to remove cumulus cells. Fertilization was defined as 2 h after the initiation of culture in the HTF medium. The cells were subsequently transferred to a drop of M16 medium for *in vitro* culture.

### Immunofluorescence and confocal microscopy

Primary antibodies included anti-YAP1 (H00010413-M01, monoclonal, 1:100 dilution; Novus Biologicals), anti-CDX2 (ab76541, monoclonal, 1:200 dilution; Abcam), anti-Ezrin (#3145, polyclonal, 1:200 dilution; Cell Signaling Technology), phospho-Ezrin (Thr567)/Radixin (Thr564)/Moesin (Thr558) (#3141, polyclonal, 1:100 dilution; Cell Signaling Technology), and rabbit anti-SOX2 (ab92494, monoclonal, 1:1000 dilution; Abcam). The secondary antibodies used were as follows: Alexa Fluor 488 goat anti-rabbit IgG Cross-Adsorbed (A11008, polyclonal, 1:400 dilution; Invitrogen), Alexa Fluor 555 goat anti-rabbit IgG (A21428, polyclonal, 1:400 dilution; Invitrogen), Alexa Fluor 488 goat anti-mouse IgG (A11001, polyclonal, 1:400 dilution; Invitrogen), and Alexa Fluor 555 goat anti-mouse IgG (A21422, polyclonal, 1:400 dilution; Invitrogen).

Oocytes and embryos were fixed using 4% (w/v) paraformaldehyde (Wako Pure Chemical Industries) in PBS for 60 min and permeabilized for 60 min with 0.2% (v/v) Triton X-100 in PBS. Next, the oocytes and embryos were blocked for 45 min with a blocking buffer (Blocking One, 1:5; Nacalai Tesque, Inc) diluted in 0.05% (v/v) Tween-20 in PBS.

Thereafter, oocytes or embryos were incubated with the primary antibody in 0.01% (v/v) Tween-20 and 5% (v/v) Blocking One in PBS or Can Get Signal solution A (TOYOBO) for 8 to 24 h at 4 °C. We used Can Get Signal solution A for diluting phospho-Ezrin (Thr567)/Radixin (Thr564)/Moesin (Thr558) and YAP1 antibodies.

After washing for 5 to 10 min in PBS containing 0.1% (v/v) Triton X-100 and 0.3% (w/v) bovine serum albumin (Sigma–Aldrich), the oocytes and embryos were incubated for 30 min at 25 °C with secondary antibodies diluted to 1:400 in 0.01% (v/v) Tween-20 and 5% (v/v) Blocking One in PBS. Nuclei were counterstained with 25 mg/ml Hoechst 33342 (Sigma–Aldrich) prepared in 0.2% (w/v) polyvinyl alcohol in PBS. Fluorescence signals were visualized using a Leica TCS SP5 confocal laser-scanning microscope (Leica).

### Microinjection of mNF2-GFP mRNA

We microinjected bovine one-cell embryos with mNF2-GFP mRNA, used in our previous report (14). A solution of 200 ng/μl purified RNA diluted with injection buffer (10 mM Tris–HCl [pH 7.4] and 0.1 mM EDTA) was microinjected into one-cell stage embryos using a FemtoJet injection device (Eppendorf). Fluorescence signals were visualized using a Leica TCS SP5 confocal laser-scanning microscope (Leica). The mNF2-GFP signal was detectable until the D5.5 morula stage but was no longer observed after D6.

### Preparation of zona-free embryos and embryos with half the number of cells

We used a D1.5 two-cell embryo for the removal of zona pellucida, separation of blastomere, and destruction of one of the two blastomeres. The zona pellucida of two-cell embryos was removed by treating with 0.02% (w/v) proteinase K for 2 min (Fuji Film Wako) in SOF medium and gentle pipetting using a glass capillary with a diameter equivalent to that of bovine embryos. After removing the zona pellucida, we washed the embryos in SOF medium and cultured them individually in small 10 μl drops. For blastomere separation, we washed the embryos in 0.2% (w/v) polyvinyl alcohol in PBS by gentle pipetting and divided them into two blastomeres after removing the zona pellucida. Next, we washed and cultured them as zona pellucida–free embryos. We destroyed one of the two blastomeres by thrusting one side of the two blastomeres approximately five times using a Femto Tip (Eppendorf).

### Imaging and statistical analyses

To analyze YAP1 fluorescence intensity, the average fluorescence intensity in the nucleus and immediately outside the nucleus (*i.e.*, the cytoplasm) was measured using the ImageJ software (National Institutes of Health). The N/C ratio of YAP1 was determined for each cell. All statistical analyses were performed using the GraphPad Prism 10 software (GraphPad). Statistical differences between two samples were analyzed using the Student’s *t* test. Statistical differences among three or more samples were analyzed using one-way ANOVA followed by Dunn’s multiple comparison test. The correlation coefficient between the cell number and the ratio of cells in which YAP1 was localized to the nuclei or cytoplasm to total cells was calculated using nonparametric Spearman’s rank correlation.

## Data availability

All data are contained within the article and/or in the supporting information.

## Supporting information

This article contains supporting information.

## Conflict of interest

The authors declare that they have no conflicts of interest with the contents of this article.
