# Supplementary material for: Polarization-independent regulation of the subcellular localization of Yes-associated protein 1 during preimplantation development
Source: J Biol Chem. 2025 Mar 19;301(4):108429. doi: 10.1016/j.jbc.2025.108429 (PMC12018982; doi:10.1016/j.jbc.2025.108429)
Supplement: Supporting information [file mmc1.pdf]

Supporting figure legends

Fig. S1. Ratio of cells classified based on YAP1 localization at multiple time points and in other sections of embryos, related to Figure 1.

(A) Immunostaining of YAP1 in other sections of the bovine morula in Fig. 1A. Scale bars, 50 µm. Z indicates the sectional number of the image. (B) YAP1 N/C ratio in YAP1 N/C/E-classified cells (N = 3, n = 74). Based on this analysis, the thresholds of the N/C ratio analyzed in the following experiments were defined as follows, (≥1.2: YAP1 N; 0.85–1.2: YAP1 E; and <0.85: YAP1 C). (C) Relationship between the ratio of YAP1 E cells to the total cell number in an embryo and total cell number in bovine morula. (D) Localization of YAP1 in the outer cells of D6 morula. (E) Immunostaining of YAP1 in mouse morula (E2.75, N = 12; E3.25, N = 19; E3.5, N = 4). Scale bars, 50 µm. (F) Relationship between YAP1 N ratio and total cell number in mouse morula. (G) Relationship between the YAP1 C ratio and total cell number in mouse morula. Capital "N" indicates the number of embryos and small letter "n" indicates the number of cells.

Fig. S2. Lower YAP1 N ratio in D6.5 bovine morula contributes less TE in D6.5 blastocyst.

(A) Immunostaining of CDX2 in D6.5 early, D8 middle bovine blastocyst, and E3.5 mouse blastocyst. Scale bars, 50 µm. (B) Comparisons of CDX2-positive ratio among D6.5, D8 bovine blastocyst, and E3.5 mouse blastocyst. [a-b]Different figures indicate statistical significance ($p < 0.05$). (C) Comparison of the YAP1 N ratio between bovine D6.5 (N = 10) and mouse E3.5 (N = 3) morulae. Asterisks represent significant differences ($p < 0.05$). Statistical differences between samples were analyzed using one-way ANOVA followed by

Dunn's multiple comparison test (B) or Student's *t*-test (C). Data are represented as the mean ± SD (B, C).

Fig. S3. Localization of p-ERM, related to Figure 3.

(A) Immunostaining of P-ERMs in bovine morula on D5 (N = 5), D6 (N = 4), and D6.5 (N = 5). (B) Localization of mouse NF2-GFP (mNF2-GFP) in bovine D5 (N = 3) and D5.5 (N = 6) morulae. Scale bars, 50 μm.

Fig. S4. Illustration of Figure 5 and YAP1 N and C ratio in HA embryos with over 32 cells, related to Figure 4.

(A) Illustration of the preparation of the three types of embryos. (B) Immunostaining of EZRIN in ZPF morula (N = 4). (C) Immunostaining of YAP1 in HA D6 and with >32-cell embryo (N = 5). (D) Comparison of the YAP1 N ratio among intact embryos with 48–64 cells, HA with 24–32 cells, and HA with >32 cells embryos. [a-b]Different figures indicate statistical significance (p < 0.05). (E) Comparison of the YAP1 C ratio among intact embryos with 48–64 cells, HA embryos with 24–32 cells, and HA embryos with >32 cells in the late morula stage (48–64 cells). Different letters indicate significant difference (p < 0.05). [a-b]Different figures indicate statistical significance (p < 0.05). Statistical differences were analyzed using the one-way ANOVA followed by Dunn's multiple comparison test (D, E). Scale bars, 50 μm (B, C).

Fig. S5. Expression of YAP1 and SOX2 in bovine embryos in morula stages, related to Figure 5.

(A) Immunostaining of YAP1 and SOX2 in bovine morula on D5 (N = 5), (B) bovine D6 morula (N = 3), and (C) bovine D6 ZPF morula (N = 4). Scale bars, 50 μm.

Fig. S1

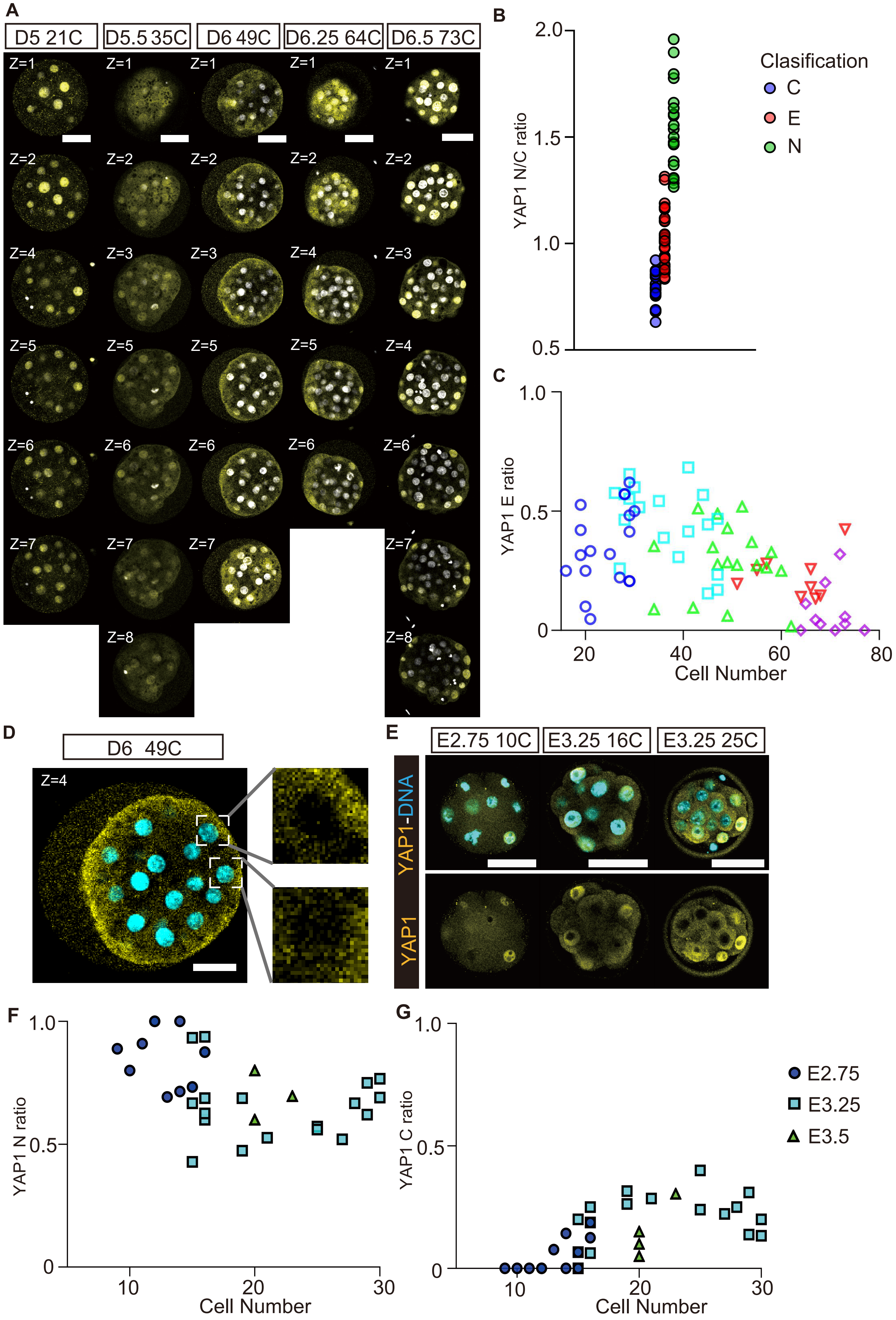
A
D5 21C
D5.5 35C
D6 49C
D6.25 64C
D6.5 73C
Z=1
Z=2
Z=3
Z=4
Z=5
Z=6
Z=7
Z=8
B
YAP1 N/C ratio
2.0
1.5
1.0
0.5
Clasification
C
E
N
C
YAP1 E ratio
1.0
0.5
0
20
40
60
80
Cell Number
D
D6 49C
Z=4
E
E2.75 10C
E3.25 16C
E3.25 25C
YAP1-DNA
YAP1
F
YAP1 N ratio
1.0
0.5
0
10
20
30
Cell Number
G
YAP1 C ratio
1.0
0.5
0
10
20
30
Cell Number
E2.75
E3.25
E3.5

Fig. S2

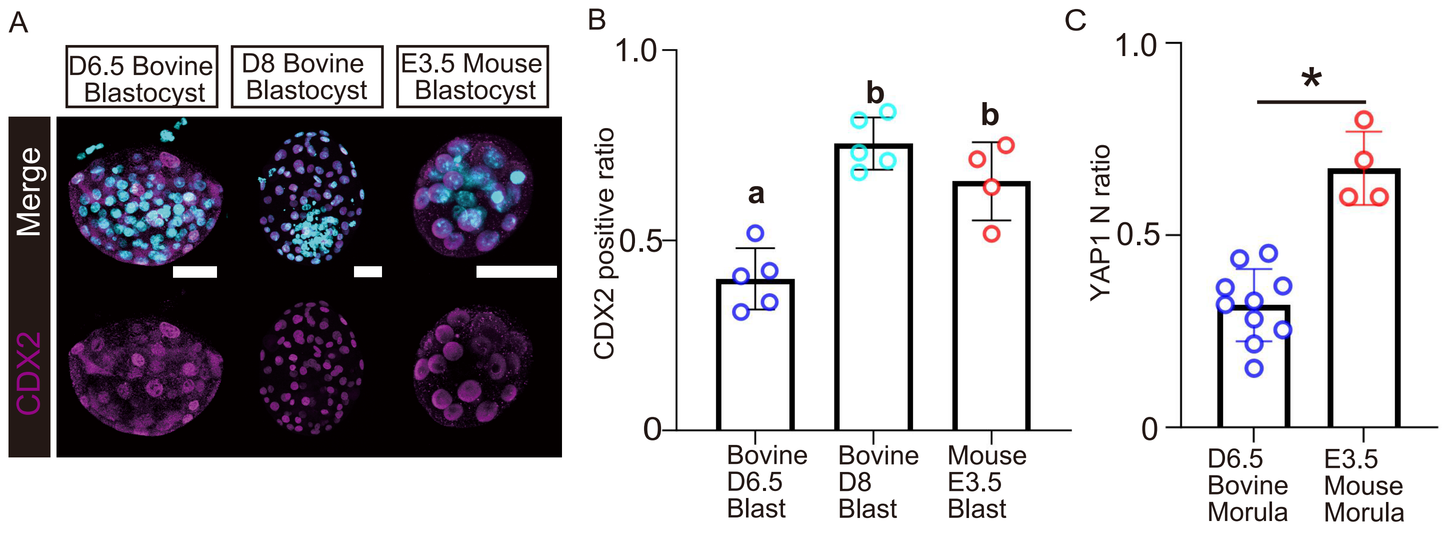
A
D6.5 Bovine Blastocyst
D8 Bovine Blastocyst
E3.5 Mouse Blastocyst
Merge
CDX2
B
CDX2 positive ratio
1.0
0.5
0
a
b
b
Bovine D6.5 Blast
Bovine D8 Blast
Mouse E3.5 Blast
C
YAP1 N ratio
1.0
0.5
0
*
D6.5 Bovine Morula
E3.5 Mouse Morula

Fig. S3

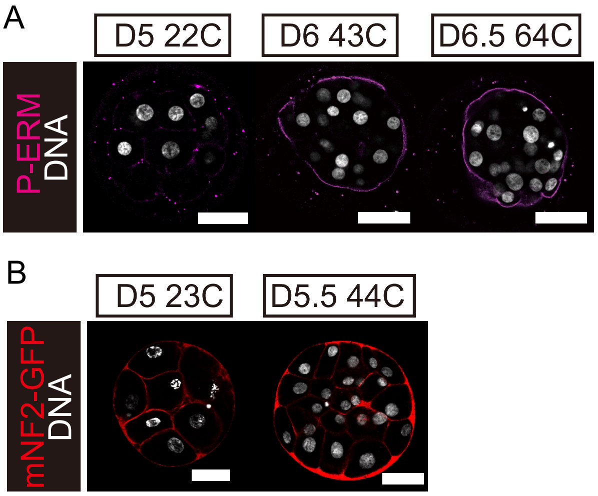

Fig. S4

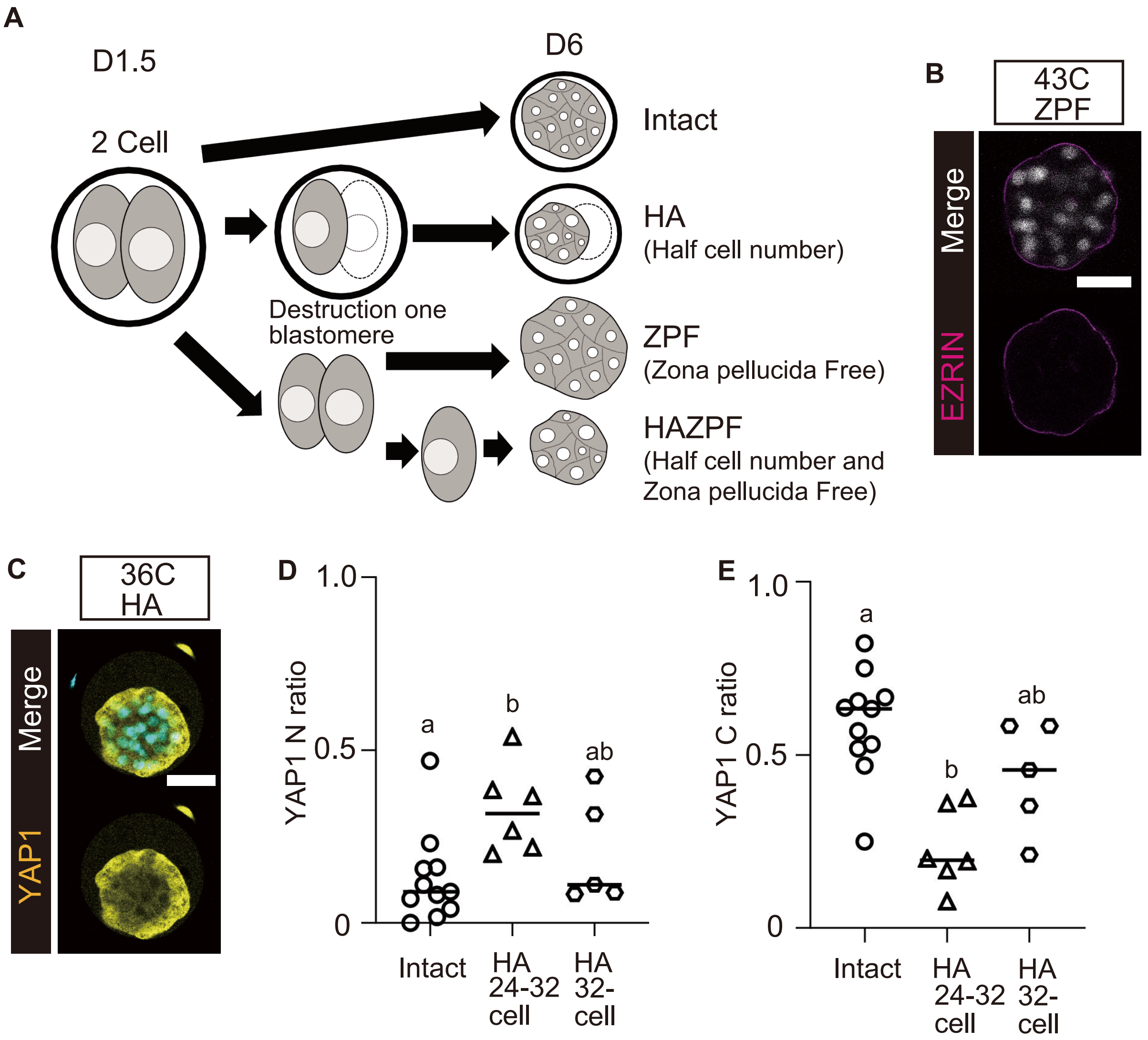

A
D1.5
D6
2 Cell
Intact
HA
(Half cell number)
Destruction one
blastomere
ZPF
(Zona pellucida Free)
HAZPF
(Half cell number and
Zona pellucida Free)
B
43C
ZPF
Merge
EZRIN
C
36C
HA
Merge
YAP1
D
YAP1 N ratio
1.0
0.5
0
a
b
ab
Intact
HA
24-32
cell
HA
32-
cell
E
YAP1 C ratio
1.0
0.5
0
a
b
ab
Intact
HA
24-32
cell
HA
32-
cell

Fig. S5

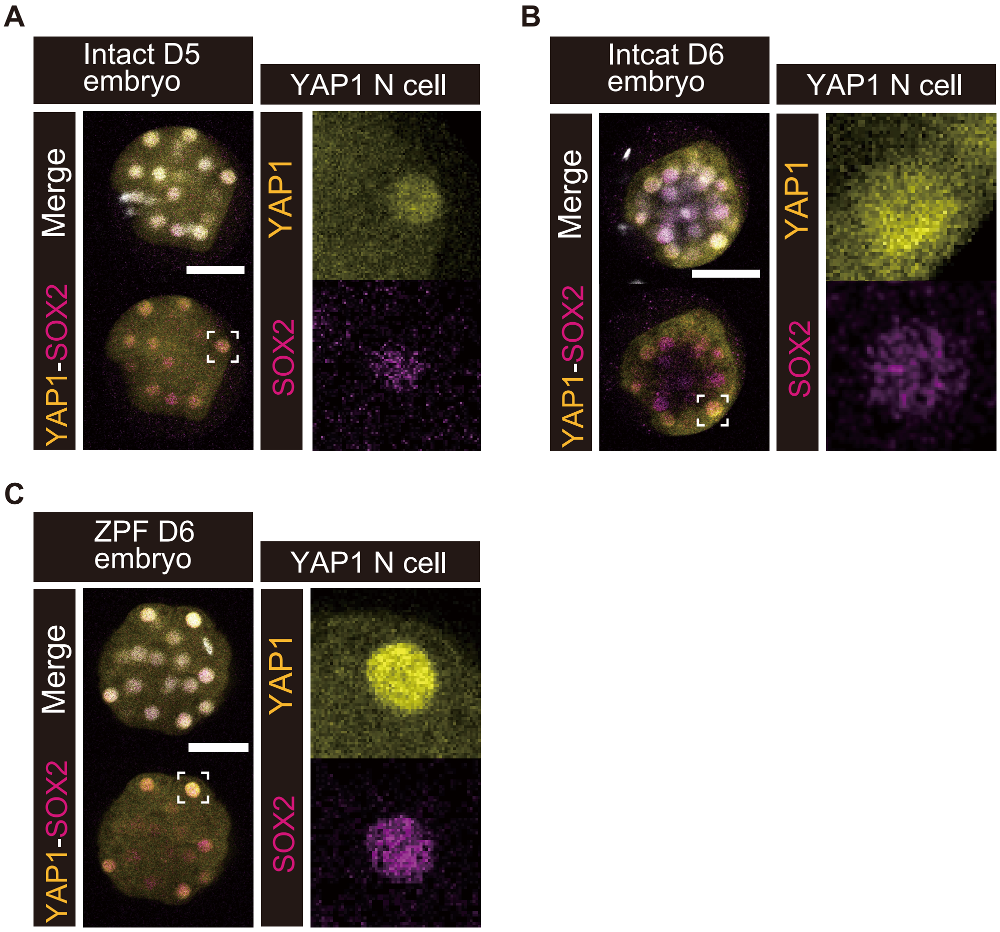
A
Intact D5 embryo
YAP1 N cell
Merge
YAP1-SOX2
YAP1
SOX2
B
Intcat D6 embryo
YAP1 N cell
Merge
YAP1-SOX2
YAP1
SOX2
C
ZPF D6 embryo
YAP1 N cell
Merge
YAP1-SOX2
YAP1
SOX2
